# Cerebral Correlates of Emotional and Action Appraisals During Visual Processing of Emotional Scenes Depending on Spatial Frequency: A Pilot Study

**DOI:** 10.1371/journal.pone.0144393

**Published:** 2016-01-12

**Authors:** Aurélie Campagne, Benoit Fradcourt, Cédric Pichat, Monica Baciu, Louise Kauffmann, Carole Peyrin

**Affiliations:** 1 Univ Grenoble Alpes, Laboratoire de Psychologie et Neurocognition (LPNC), Grenoble, France; 2 Centre national de Recherche Scientifique, LPNC, Grenoble, France; Centre de Neuroscience Cognitive, FRANCE

## Abstract

Visual processing of emotional stimuli critically depends on the type of cognitive appraisal involved. The present fMRI pilot study aimed to investigate the cerebral correlates involved in the visual processing of emotional scenes in two tasks, one emotional, based on the appraisal of personal emotional experience, and the other motivational, based on the appraisal of the tendency to action. Given that the use of spatial frequency information is relatively flexible during the visual processing of emotional stimuli depending on the task’s demands, we also explored the effect of the type of spatial frequency in visual stimuli in each task by using emotional scenes filtered in low spatial frequency (LSF) and high spatial frequencies (HSF). Activation was observed in the visual areas of the fusiform gyrus for all emotional scenes in both tasks, and in the amygdala for unpleasant scenes only. The motivational task induced additional activation in frontal motor-related areas (e.g. premotor cortex, SMA) and parietal regions (e.g. superior and inferior parietal lobules). Parietal regions were recruited particularly during the motivational appraisal of approach in response to pleasant scenes. These frontal and parietal activations, respectively, suggest that motor and navigation processes play a specific role in the identification of the tendency to action in the motivational task. Furthermore, activity observed in the motivational task, in response to both pleasant and unpleasant scenes, was significantly greater for HSF than for LSF scenes, suggesting that the tendency to action is driven mainly by the detailed information contained in scenes. Results for the emotional task suggest that spatial frequencies play only a small role in the evaluation of unpleasant and pleasant emotions. Our preliminary study revealed a partial distinction between visual processing of emotional scenes during identification of the tendency to action, and during identification of personal emotional experiences. It also illustrates flexible use of the spatial frequencies contained in scenes depending on their emotional valence and on task demands.

## Introduction

Emotional stimuli (e.g. scenes representing dangerous or friendly animals, emotional faces) are generally detected and identified faster than neutral stimuli [1–4], suggesting that the intrinsic emotional properties of visual stimuli facilitate their perceptual processing. On a cerebral level, this facilitation results in greater activation of visual regions by emotional stimuli than by neutral stimuli [5–9]. A similar pattern is also observed for the amygdalae which are specifically involved in emotional processing, and may be partially responsible for the modulation by emotions of activity in the visual regions due to direct and reciprocal connectivity between the two regions [10–16].

Visual processing of emotional information is also modulated by task demands [1,11]. Various tasks involving different types of cognitive appraisal have been used in the literature, and may be grouped in terms of affective and non-affective tasks. Affective tasks explicitly involve emotional processes, and include cognitive tasks evaluating, for example, personal emotional experiences or those of others in response to emotional stimuli. Non-affective tasks are unrelated to emotional processes and include cognitive tasks such as identifying the gender of emotional faces or counting people in an emotional scene (in this type of task, any emotional processing involved would be implicit). Some fMRI studies have shown that affective tasks induce greater activity in visual regions and in the amygdala than non-affective tasks [17,18]. Similar results have been reported in EEG studies, where higher LPP amplitude–an event-related potential whose origin is located in occipital and infero-temporal and parietal areas–has been observed during affective tasks [19–23]. Several authors suggest that these task-demand based modulations of activity in visual regions might be closely related to activity in prefrontal structures such as the medial and lateral prefrontal cortices and the orbitofrontal cortex [24–28].

In the majority of studies, the effect of affective appraisals on the visual processing of emotional stimuli is assessed using tasks which require the identification of a personal emotional experience [29,30] or the emotions of others [31,32]. However, visual processing of emotional stimuli may also be modulated by two motivational systems, one defensive and the other appetitive. These motivational systems stimulate individuals to act and respond with adapted behaviors and tendencies to action using either avoidance (defensive system) or approach (appetitive system) [33–37], which are preferentially adopted for unpleasant (except for anger stimuli, [38]) and pleasant stimuli respectively. Compared to the cerebral network recruited by the appraisal of personal emotional experience, relatively little is known about the network recruited by the appraisal of tendency to action. To our knowledge, no study has as yet investigated and compared the neural bases of these two types of appraisals during the visual processing of emotional stimuli.

Interestingly, cognitive evaluation of visual stimuli has been shown to be driven by a specific spatial frequency content [32,39–41]. Recent behavioral and neuroimaging studies [31,42–50] and computational data [51,52] suggest that emotional processing of visual stimuli may rely on the rapid processing of low spatial frequency information (LSF), especially in the case of threat. However, some behavioral studies report a relative flexibility in the use of spatial frequency information during the visual processing of emotional stimuli depending on a task’s demands [1,32,41,53]. Schyns and Oliva [32], for instance, show that high spatial frequency (HSF) information is preferentially used for deciding whether a face is expressive or not, whereas LSF information is preferred for the identification of a specific emotion (happiness, anger or neutrality). Using natural scenes, Fradcourt et al. [1] found that HFS information was the most relevant in the rapid identification of a personal emotional experience (unpleasant, pleasant or neutral), while LSF information was required for rapid identification of the tendency to action (avoidance, approach or no action). This last study confirmed the importance of dissociating motivational appraisal based on tendency to action from emotional appraisal based on personal emotional experience, and of studying the role of spatial frequencies in the brain structures involved in each of these two appraisals during the visual processing of emotional stimuli.

The present fMRI pilot study aimed to identify the cerebral correlates involved in the processing of emotional scenes during two affective tasks, one “emotional” based on subjective emotion and the other motivational based on action tendency. The effect of spatial frequencies of visual stimuli on this cerebral network was subsequently explored for each task by using scenes filtered in LSF and HSF, and non-filtered scenes. In the emotional task, participants were instructed to indicate their *emotional experience* relating to each scene (unpleasant, pleasant or neutral). In the motivational task, they had to determine their spontaneous *tendency to action* for each scene (avoidance, approach or no action). Importantly, we used exactly the same paradigm for both tasks, in order to investigate the influence of the cognitive demands of the task, irrespective of low-level visual processing.

According to the majority of theorists, the function of emotion is to prepare and motivate the organism to deal with environmental requirements. This suggests that emotion, motivation and action tendency are closely linked. The “subjective feeling” and “motivational relevance” of emotion therefore both contribute to the salience of emotional stimuli. Based on this point of view, we hypothesized an overlap of activation in certain brain regions involved in the emotional and motivational tasks. Greater activity was expected in the amygdala and visual regions during the visual processing of emotional stimuli (versus neutral stimuli) in both tasks. The amygdala plays an important role in emotional processing and motivational processes and has direct and reciprocal connections with visual cortices [10–16]. During visual processing, we also hypothesized activation of prefrontal regions such as the ventrolateral prefrontal cortex in the emotional task and the dorsolateral prefrontal cortex in the motivational task given their respective suggested roles in the verbal labelling of emotions [54], and in directing action tendency [38,55,56]. Activation of the ventromedian prefrontal cortex and the orbitofrontal cortex was also expected in both tasks, as these regions participate in the assessment of the emotional value of stimuli prior to the selection of appropriate actions [57–59]. Finally, we hypothesized that activity in all these regions may be modulated by the spatial frequency content of scenes depending on the task’s demands. In particular, we expected to observe greater activity in the amygdala and visual and prefrontal regions for scenes filtered in HFS than for scenes filtered in LFS in the emotional task, and expected that the opposite pattern would emerge in the motivational task, based on previous studies [1, 49] and on neuroanatomical and functional data on the pathways involved in visual processing for action [60, 61].

## Method

### Participants and ethics statement

Thirteen participants (7 men and 6 women; mean age: 22 ± 2 years), all right-handed, were selected for the experiment. They had normal or corrected-to-normal vision; participants requiring visual correction wore the MediGoggle Adult Research Set (Cambridge Research Systems Ltd, England; http://www.crsltd.com/), interchangeable prescriptive goggles suitable for use in MR environments. No participant was taking any medical treatment likely to modulate the emotional processes (e.g. beta-blockers) or had any history of neurological or psychiatric disease.

All participants gave their informed written consent before participating in the study, which was carried out in accordance with The Code of Ethics of the World Medical Association (Declaration of Helsinki) for experiments involving humans, and approved by the local ethics committee (Comité de protection des personnes Sud-Est V, France, n° RCB: 2011-A01536-35) and the local health safety agency.

### Experimental procedure

#### Stimuli and procedure

Stimuli consisted of 162 black and white photographs of natural scenes, all 640 x 480 pixels in size (with a visual angle of 31 x 24°), and were composed of 54 pleasant, 54 unpleasant and 54 neutral scenes. Visual scenes were in close-up and directly involved the participant (i.e. first-person perspective). Pictures were selected from several sources: the International Affective Picture System (IAPS; [62]), the Internet (no copyrighted material) and an in-house database. Unpleasant scenes included dangerous animals (sharks, snakes, spiders, etc.), unsafe environments (tornadoes, fires, tsunamis, etc.) and aggressive people (carrying weapons, with angry expressions, etc.). Pleasant scenes included images showing safe, happy and friendly animals, idyllic landscapes (beaches, mountains, etc.) as well as happy and friendly people. Neutral scenes included the same type of stimuli (animals, environments and people) in neutral situations.

Stimuli were selected from an in-house database created for, and used in, a previous study [1] (stimuli pretested on 108 subjects [1,6]), and were characterized by emotional valence, arousal level and tendency to action. Unpleasant stimuli (-6.43 ± 1.42; on a valence scale from 10-pleasant to -10-unpleasant with 0- absence of or weak valence) induced an arousal level of 5.51 (± 0.93; on a scale from 1-low arousal to 10-high arousal) and a tendency to avoid (-7.27 ± 1.18; on an action tendency scale from 10-approach to -10-avoid with 0- absence of or weak tendency to action). Pleasant stimuli (5.11 ± 0.74 on the valence scale) induced an arousal level of 4.90 (± 0.92) and a tendency to approach (5.06 ± 1.14). Neutral scenes judged to be without emotional content (neutral valence: 0.02 ± 1.01) were characterized by a low arousal level (1.81 ± 0.97) and a weak preferential tendency to action (0.61±1.56). It should be noted that significant positive correlations were observed between the action tendency (absolute value) and the arousal level of pictures (r = 0.87, p<0.001), supporting the idea that “arousal” reflects (and could predict) the degree of motivational activation. However, a similar correlation value was observed between emotional valence and arousal (r = 0.91, p<0.001) and between action tendency and emotional valence (r = 0.96; p<0.0001) suggesting a close relationship between these three dimensions in our study.

Stimuli were created using the MATLAB image processing toolbox (Mathworks Inc., Sherborn, MA, USA). Spatial frequency content of scenes was filtered by multiplying the Fourier transform of original pictures with Gaussian filters. The standard deviation of the Gaussian filter is a function of the spatial frequency cut-off, for a standard attenuation of 3 dB. We removed spatial frequency content above 1 cycle per degree of visual angle (i.e., low-pass cut-off of 31 cycles per image) for LSF stimuli and below 6 cycles per degree (i.e., high-pass cut-off of 186 cycles per image) for HSF stimuli. The spatial frequency content of scenes was not modified in non-filtered (NF) stimuli The resulting images were then normalized to obtain a mean luminance of 0.5 (for luminance values of between 0 and 1; i.e. mean luminance of 128 on a 256 grey-scale-level) and a standard deviation of 1 (i.e. 25.5 on a grey-level scale; root mean square [RMS] contrast; see [63]).

Stimuli were displayed using E-prime software (E-prime Psychology Software Tolls Inc., Pittsburgh, USA) and back-projected onto a translucent screen positioned at the rear of the magnet. Participants viewed this screen at a distance of about 140 cm via a mirror fixed on the head coil.

**Fig 1:**
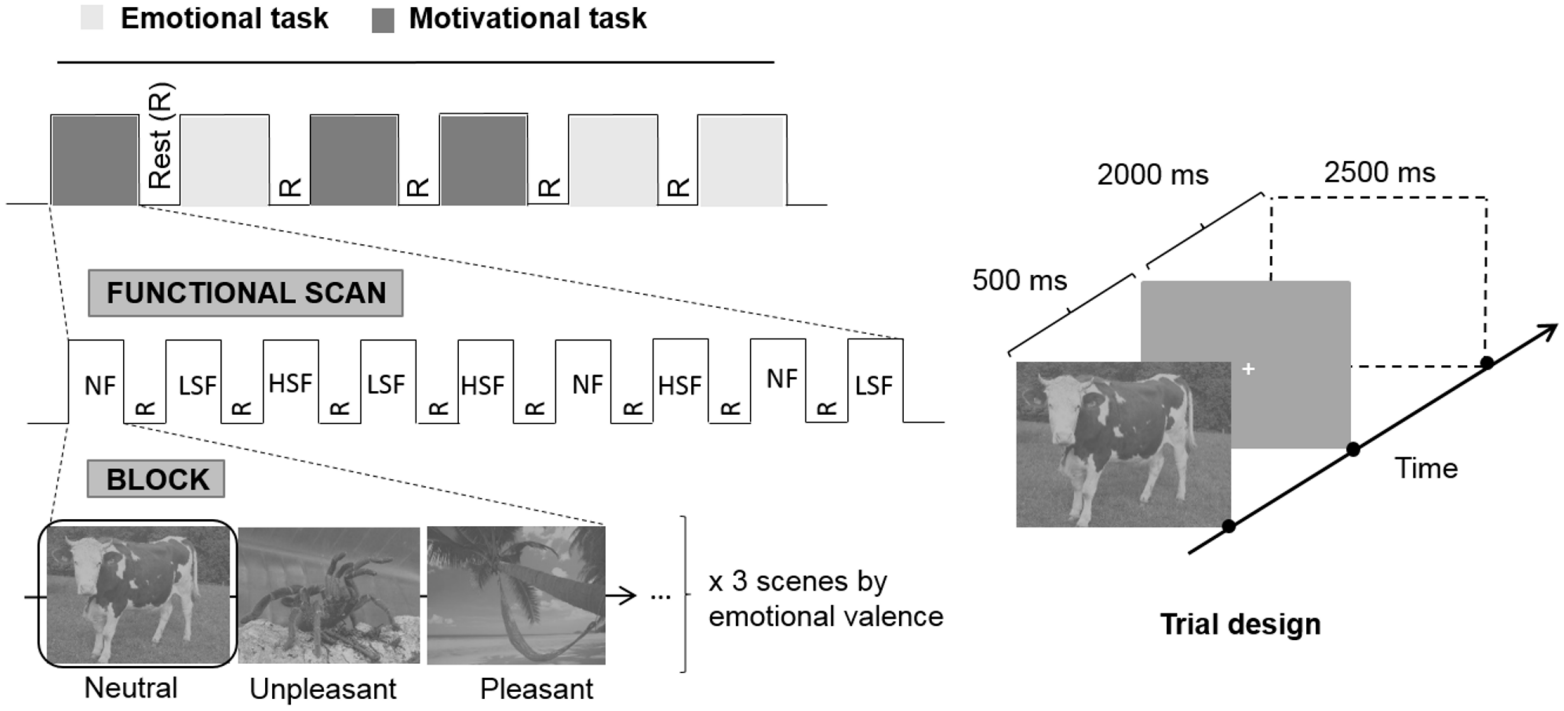
Experimental design.

The experiment consisted of six functional scans, three scans for the emotional task based on emotional experience and 3 scans for the motivational task based on tendency to action. Scan order was counterbalanced between participants. For each scan, three spatial frequency (SF) contents of natural scenes (NF, LSF and HSF) were manipulated in a block design paradigm (Fig 1). Each functional scan lasted 6.5 minutes and was composed of nine 30-sec SF-blocks (3 NF, 3 LSF, 3 HSF), separated from each other by a 15-sec block with a fixation cross in the center of the screen (fixation condition) displayed against a gray background. The order of SF-blocks was counterbalanced between scans and participants. In an SF-block, a pseudo-randomized event-related paradigm displaying 9 scenes (3 unpleasant scenes, 3 pleasant scenes, 3 neutral scenes) and 3 null-events with a fixation cross in the center of the screen against a gray background was used. The inter-stimuli duration between events was 2500 ms. In order to minimize repetition effects, each scene was presented only once in each spatial frequency condition (NF, LSF, HSF) and in the different functional scans.

For each task (across 3 fMRI scans), a total of 243 trials were presented with 27 trials per spatial frequency (3) and per emotional valence (3). These trials began with a scene being displayed for 500 ms, followed by a white fixation cross for 2000 ms, both against a gray background. It should be noted that we used a stimulus duration of 500 ms on the basis of a previous study [1] in order to guarantee the correct recognition of emotional information when complex scenes filtered in HSF and LSF were visualized. In each type of task, participants were asked to respond as quickly and as accurately as possible by pressing a key using their dominant hand as soon as a scene appeared. In the emotional task, participants were required to specify their *emotional experience*, i.e. they had to decide whether each scene was pleasant, unpleasant or neutral/no emotion by pressing the corresponding response key. In the motivational task, participants were required to indicate the action they would adopt if they were involved in the scene or their *action tendency*, i.e. in each trial, they had to decide whether they would approach, avoid or not act by pressing on the corresponding response key. Each task involved three response choices corresponding to three response keys. In both tasks, the assignment of the three emotions (emotional task) or the three action tendencies (motivational task) to the three keys was indicated to participants before the experiment and counterbalanced between participants. Participants were familiarized with each task in advance outside the scanner using training trials which were different from those used in the experiment.

Response accuracy and reaction time in milliseconds (RT, ms) were recorded for each trial. In both tasks, a response for a given scene was considered correct when the response (emotional valence or tendency to action) given by the participant was the same as the one given by participants in the database pre-test, i.e. when the response was congruent. In the emotional task, a correct response corresponded to i) an unpleasant response for pictures categorized as unpleasant during the pre-test, ii) a pleasant response for pictures categorized as pleasant during the pre-test and iii) a neutral response for pictures categorized as neutral during the pre-test. In the motivational task, a correct response corresponded to i) an avoidance response for pictures characterized by a tendency to avoidance in the pre-test, ii) an approach response for pictures characterized by a tendency to approach in the pre-test and iii) a no action response for pictures characterized by no action in the pre-test. All different responses (i.e. non congruent and key errors) were counted as errors. Only correct responses were taken into consideration when calculating mean reaction times.

### MRI acquisition

Acquisition of data was performed using a whole-body 3-T MRI Philips scanner (Philips Medical Systems, Best, NL) with a 32-channel head coil at the Grenoble MRI facility IRMaGe in France. Functional T2-weighted images were acquired using a gradient echo-planar imaging sequence (repetition time (TR) = 2.5s, time echo (TE) = 30 ms; descending acquisition; flip angle = 80°; field of view (FOV) = 220 x 216 mm2; acquisition matrix = 88 x 85 pixels; reconstruction matrix = 96 x 96 pixels; in-plane voxel size = 2.89 x 2.89 mm). Forty-four adjacent axial slices were acquired in descending mode (Slice thickness: 3.25 mm, 0 mm gap), covering the entire brain. All scanning parameters were selected to optimize the quality of the BOLD signal while maintaining a sufficient number of slices to acquire whole-brain data. In addition, for each participant, a rotation of approximately 15° was applied on the bi-commissural classical plane on the basis of an anatomical scan performed just before experiments, so that the new sectional plane connected the lower limits of the frontal lobe and of the pons. This plane was chosen to optimize the acquisition of data in temporal median regions and orbitofrontal regions. Finally, for each participant, a T1-weighted high-resolution three-dimensional anatomical volume was acquired (128 adjacent anatomical slices; ~ 1.17mm thick each; FOV = 224 x 256 x 175 mm2; acquisition matrix = 192 x 132 x 128 pixels; reconstruction matrix = 288 x 288 x 128 pixels; voxel size = 1.17 x 1.94 x 1.37 mm) and normalized to the MNI template. The orientation of anatomical slices was similar to that of the functional slices

### Data processing

#### Whole-brain analysis

Data analysis was performed using the general linear model [64] for block designs in SPM8 (Wellcome Department of Imaging Neuroscience, London, UK, www.fil.ion.ucl.ac.uk/spm) implemented in Matlab 7 (Mathworks, Inc., Sherborn, MA). Individual functional volumes were realigned, time-corrected, normalized to the MNI space and spatially smoothed by an 8-mm FWHM Gaussian kernel. Time series for each voxel were high-pass filtered (1/128 Hz cutoff) to remove low-frequency noise and signal drift.

On an individual level, statistical parametric maps were computed for several contrasts of interest. The first aim of the study was to identify and compare the cerebral regions involved in two distinct affective tasks. We therefore identified the cerebral regions specifically involved in the emotional task and the motivational task by contrasting one task to another ([ET > MT] and [MT > ET]). These analyses were performed by taking into account the individual emotional valences of visual stimuli (see contrasts in results). The second aim was to investigate the influence of spatial frequency information on the emotional processes. We contrasted the spatial frequency conditions against one another (HSF and LSF) for the emotional task ([ET LSF < ET HSF] and [ET HSF < ET LSF]) and for the motivational task ([MT LSF < MT HSF] and [MT HSF < MT LSF]). Once again, the individual emotional valences of visual stimuli in each task were taken into account when performing these analyses (see contrasts in Results). Results were reported with a voxel-wise threshold of p < 0.001 (uncorrected for multiple comparisons, T > 3.93) and a cluster extent threshold of 20 voxels. Significant activities obtained after statistical correction for multiple comparisons (FWE) with a voxel-wise threshold of p<0.05 and a cluster extent threshold of 10 voxels are also indicated in result tables. The coordinates of activated clusters were identified in MNI space.

#### Regions of interest (ROIs) in the amygdala

**Fig 2:**
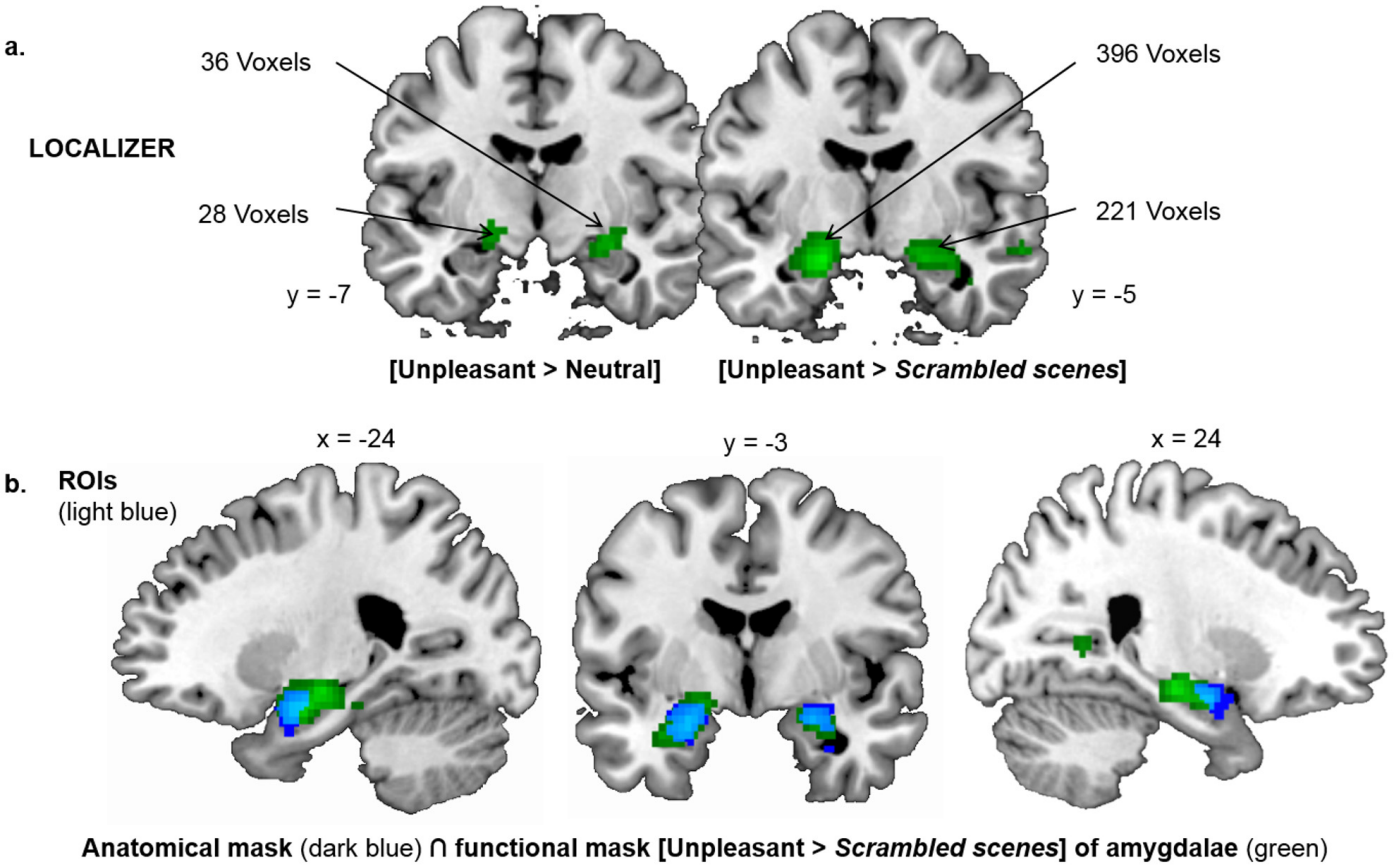
ROIs. (a) amygdala-hippocampal cluster obtained for contrasts [Unpleasant condition > Neutral condition] and [Unpleasant condition > Scrambled condition] in the localizer experiment. b) ROIs of right and left amygdala constructed from the intersection (common region in light blue) between the amygdala-hippocampal cluster obtained for the contrast [Unpleasant condition > Scrambled condition] (in green), and anatomical masks of right and left amygdala predefined from the WFU PickAtlas (in dark blue).

Previous research has linked emotional and motivational processing to activation in the amygdala [53, 65–68]. Analyses were completed by carrying out statistical comparisons of activity in the left and right amygdala as ROIs, based on the three factors of interest: task demands, emotional valence and spatial frequency content. ROIs were defined anatomically and functionally. The anatomical definition of the amygdala encompasses several nuclei with different functions [69], some of which are distinct from the neural processes examined in our study, such as classical conditioning and memory encoding and consolidation [70–72], receiving and processing pain information [73], reward processing [74]. We therefore began by identifying and delimiting ROIs using a separate functional localizer experiment performed by the same participants. The localizer experiment was adapted from previous studies and from pilot studies conducted by our team that showed activation of the amygdala. Participants viewed intact and scrambled photographs of unpleasant and neutral scenes and faces in separate blocks of a block design paradigm (see details in the next paragraph). On an individual level, the amygdalae were identified in both hemispheres by contrasting intact unpleasant stimuli (scenes + faces) to scrambled unpleasant stimuli (scenes + faces). It should be noted that the contrast between unpleasant and neutral stimuli did not allow us to identify ROIs. Two-stage random effect analyses were then performed based on individual analyses using sample t-tests. Clusters of activated voxels were then identified, based on the intensity of individual responses (p < 0.001 uncorrected for multiple comparisons, T > 3.93 with a minimum cluster extent of 20 voxels.). The localizer experiment revealed activation of the amygdala-hippocampal region in the right hemisphere (peak coordinates: 20x, -15y, -14z; 221 voxels) and the left hemisphere (-16x, -15y, -14z; 396 voxels). The functional masks were defined based on these activations. Because of the extent of activations, a standardized, predefined neuro-anatomical mask of the amygdala from the Wake Forest University (WFU) PickAtlas (Version 3.0, [75]) was also used to define ROIs. ROIs in the left and right amygdalae were thus defined by the intersection between the functional and anatomical masks (Fig 2). Parameter estimates (% signal change relative to the global mean intensity of signal) of the affective appraisal experiment were extracted from these two ROIs for each participant using in-house software. The mean percentage of signal change was calculated for each condition (Tasks–*Emotional*, *Motivational* x Spatial Frequency–*NF*, *LSF*, *HSF* x Emotional Valence–*Unpleasant*, *Pleasant*, *Neutral*). These values were subjected to a repeated-measures ANOVA using Statistica 10.0 software, with Task, Spatial Frequency, and Emotional Valence as within-subjects factors, with a statistical threshold of p < 0.001.

Stimuli used in the localizer experiment were different from the main experiment and included black and white photographs of 52 natural scenes and 52 facial expressions selected from a home database (partially published, [6]) and the KDEF database (Karolinska Directed Emotional Face set: [76]). Half of the scenes and faces were unpleasant (scenes: -6.41 ± 1.33; faces: 5.59 ± 1.02 on a scale from 10-pleasant to -10-unplesant with 0-absence of or weak valence) and induced high arousal levels (scenes: 5.68 ± 0.93; faces: 3.62 ± 0.99 on a scale from 1-low arousal to 10-high arousal), the other half was judged neutral (scenes: 0.35 ± 1.26; faces: 0.75 ± 0.58) and induced low arousal levels (scenes: 0.02 ±1.01; faces: 1.57 ± 0.35). The experiment also included scrambled pictures of all stimuli; they were created by dividing and randomizing intact picture scenes and faces into 20 × 20 pixel squares. Based on the literature [4,49,74,77,78], unpleasant scenes (threatening animals) and facial expressions (fear) were chosen in order to maximize activation of the amygdala. Neutral and scrambled stimuli corresponded to control conditions commonly used in the topic of our study [79]. All stimuli were sized 560 x 480 pixels (with a visual angle of 27° x 23°) and their luminance and RMS contrast were normalized. The localizer experiment consisted of two functional scans. Each functional scan lasted 6 min and was composed of twelve 15-sec task blocks (two blocks for each stimulus type) including 15 different photographs of the same type, separated from each other by a 15-sec block with a fixation cross in the center of the screen displayed against a gray background. Participants performed a “1-back” repetition detection task. They were instructed to press a key whenever they saw two identical stimuli repeated. This task ensured that participants paid at least as much attention to unmotivating stimuli (e.g., scrambled images) as to more interesting stimuli (scenes and faces). Only two repetitions were presented per block. Each stimulus was presented for 500 msec, with a 500-msec ISI during which a fixation cross was displayed in the center of the screen.

## Results

### Behavioral results

Mean reaction times (mRT) and accuracy for each experimental condition during the affective appraisal experiment are presented in Table 1. mRT and mean %ACC were entered in a 2 x 3 x 3 mixed design ANOVA with Task (emotional appraisal, motivational appraisal), Emotional valence (unpleasant, pleasant, neutral) and Spatial frequency (NF, LSF, HSF) as within-subject factors. Mean comparisons were explored using planned comparisons and Tukey post-hoc test. The test’s level of significance was set at 0.05.

**Table 1:** Mean reaction times (mRT) and accuracy (%ACC) obtained for each emotional valence and spatial frequency condition during the emotional and motivational tasks.

		Emotional appraisal task			Motivational appraisal task		
		Unpleasant	Pleasant	Neutral	Unpleasant (avoidance)	Pleasant (approach)	Neutral (no action)
**NF**	**RT(ms)**	792.44	820.70	1093.70	838.27	877.14	1098.58
	*ER*	± 26.51	± 19.66	±19.90	± 31.56	± 21.75	± 40.23
	**%ACC**	92.88	90.03	62.96	88.61	84.33	61.54
	*ER*	± 2.20	± 3.29	± 3.51	± 3.21	± 5.09	± 5.92
**LSF**	**RT(ms)**	820.54	878.30	1048.95	845.24	916.76	1099.53
	*ER*	± 26.99	± 23.24	± 33.31	± 30.57	± 19.05	± 41.29
	**%ACC**	88.03	86.61	62.39	88.32	80.34	63.25
	*ER*	± 1.97	± 6.62	± 4.61	± 2.96	± 5.60	± 4.97
**HSF**	**RT(ms)**	833.36	910.40	1045.93	885.84	914.09	1088.97
	*ER*	± 26.99	± 18.00	± 34.88	± 39.23	± 20.48	± 37.44
	**%ACC**	88.03	85.18	61.82	82.34	79.77	64.39
	*ER*	± 1.83	± 4.48	± 4.45	± 3.49	± 4.62	± 5.06

The ANOVA conducted on %ACC revealed that accuracy was only statistically modulated by Emotional valence (F2,24 = 19.39; p< 0.001; η2 = 0.62), Spatial frequency (F2,24 = 7.10; p < 0.01; η2 = 0.37). Planned comparisons showed better %ACC for unpleasant (F1,12 = 46.20; p < 0.001) and pleasant (F1,12 = 13.24; p < 0.01) scenes than for neutral scenes. No significant difference was noted between unpleasant and pleasant scenes (F1,12 = 1.55; p = 0.24). In addition, %ACC was better for NF scenes than for HSF scenes (F1,12 = 13.92; p < 0.01) but no distinction was noted between NF and LSF scenes (F1,12 = 3.61; p = 0,08) or between HSF and LSF scenes (F1,12 = 3.80; p = 0.07).

The ANOVA conducted on mRTs revealed a main effect of Task (F1,12 = 5.86; p < 0.05; η2 = 0.33). Shorter mRT was observed in the emotional task (916.03 ± 23.98 ms) than in the motivational task (952.82 ± 25.56 ms). mRT was also significantly modulated by Emotional valence (F2,24 = 53.48; p < 0.001; η2 = 0.82), Spatial frequency (F2,24 = 6.71; p < 0.005; η2 = 0.36) and by the interaction Emotional valence x Spatial frequency (F4,48 = 5.74; p < 0.001; η2 = 0.33). The interaction Task x Emotional valence x Spatial frequency also tended towards significance (F4,48 = 2.73; p = 0.06; η2 = 0.16). Unpleasant and pleasant scenes were categorized more quickly than neutral stimuli for all tasks and all spatial frequencies (all comparisons p<0.001). Furthermore, mRTs were significantly shorter for LSF scenes than for HSF scenes only for pleasant stimuli in the emotional task (F1,12 = 5.47; p < 0.04). Finally, in both emotional and motivational tasks, NF scenes were categorized more quickly than HSF scenes for unpleasant (F1,12 = 5.31, p < 0.04; F1,12 = 6.71; p < 0.03) and pleasant stimuli (F1,12 = 23.38, p < 0.001; F1,12 = 6.26; p < 0.03), and more quickly than LSF scenes for pleasant stimuli (F1,12 = 5.46, p < 0.04; F1,12 = 12.35; p < 0.005.

### fMRI results

#### Brain regions involved in emotional and motivational tasks

**Fig 3:**
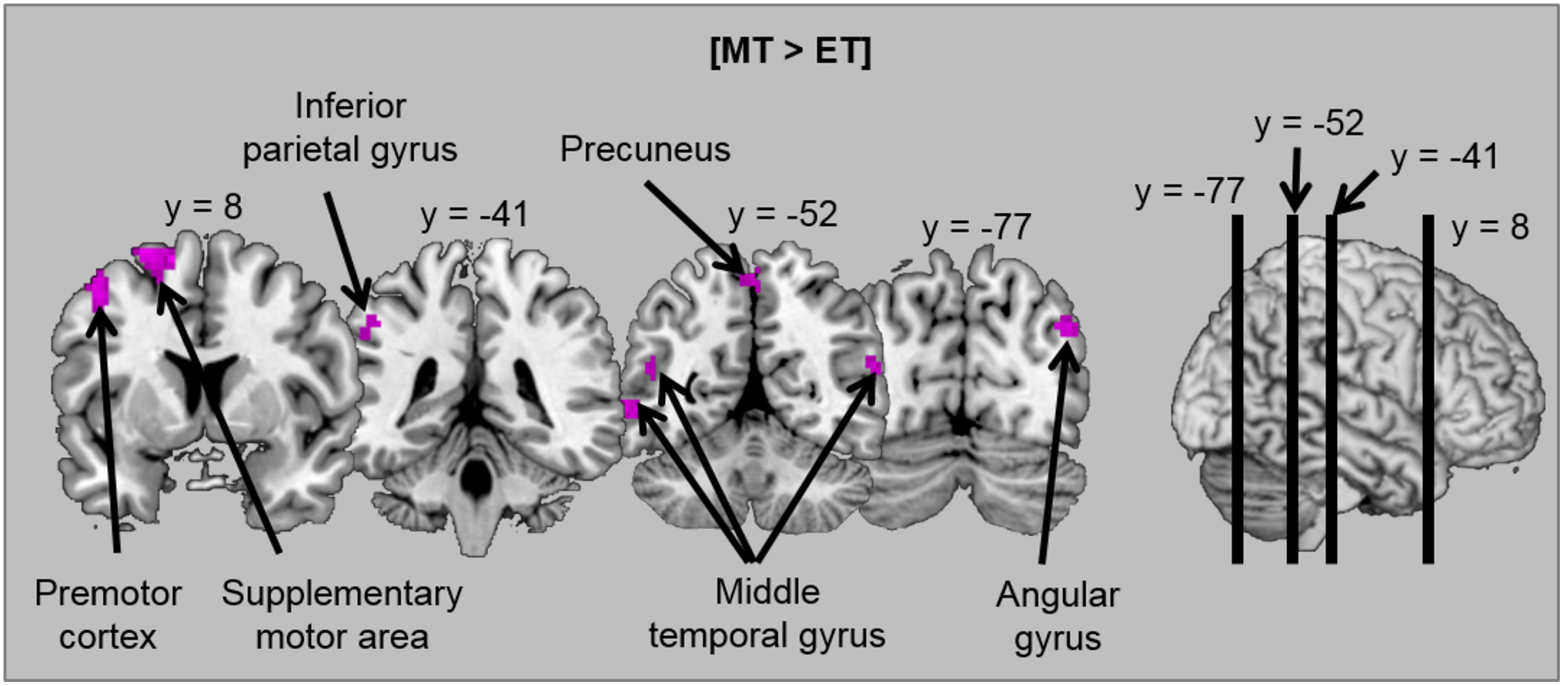
Illustration of activations obtained when the motivational task was contrasted against the emotional task [MT > ET].

Firstly, the cerebral regions specifically involved in emotional and motivational tasks were assessed by contrasting the tasks against one another ([ET > MT] and [MT > ET]). Brain activations are illustrated in Table 2 and Fig 3. In comparison to the emotional task, the motivational task activated a broad fronto-temporo-parietal network. Temporal regions included the bilateral middle temporal gyrus. Parietal activations extended over the precuneus bilaterally, the right angular gyrus and the left inferior parietal gyrus. Finally, frontal regions included a part of the left middle and superior frontal gyri in the premotor cortex and supplementary motor area. No specific activations were observed for the emotional task in comparison to the motivational task.

**Table 2:** Cerebral regions involved in emotional (ET) and motivational (MT) tasks when tasks were contrasted against one another ([ET > MT] and [MT > ET]).

Cerebral regions	[MT>ET]	
	(x,y,z)	k
**Temporal lobe**		
Middle temporal gyrus	L -56;-67;19	85
**Parietal lobe**		
Angular gyrus	R 45;-75;31	27
Inferior parietal gyrus	L -46;-40;43	21
Precuneus	L/R 2;-50;58	45
**Frontal lobe**		
Middle frontal gyrus	L -46;8;52	57
Superior frontal gyrus	L -26;8;67	150

The MNI coordinates (x, y, z), the cluster extent (k = number of voxels) and the laterality (Left-L and Right-R) were indicated for each activation cluster. Results were reported with a vowel-wise threshold of p<0.001 (uncorrected for multiple comparisons, T > 3.93) and a cluster extent threshold of 20 voxels. It should be noted that no specific activation was observed for the contrast [ET > MT].

**Fig 4:**
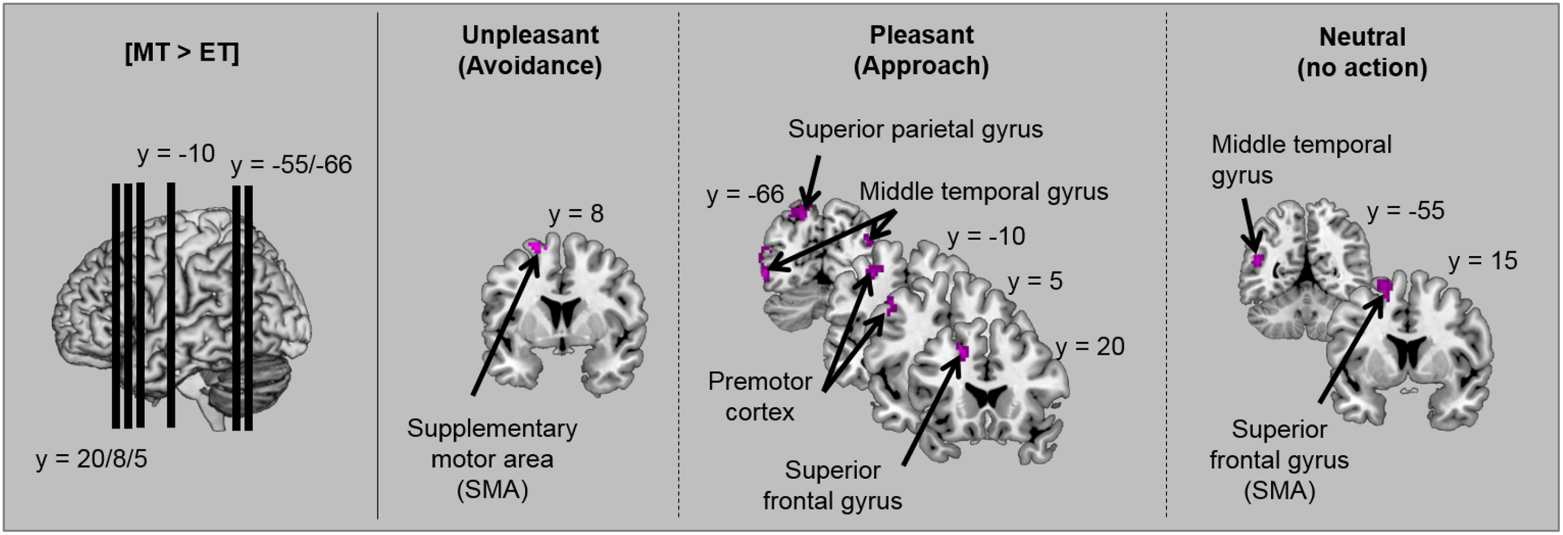
Illustration of activations obtained when the motivational task was contrasted against the emotional task [MT > ET] for each emotional valence.

In order to investigate the extent to which specific activations for the task depend on the emotional valence of stimuli, the contrasts between tasks ([ET > MT] and [MT > ET]) were replicated by distinguishing unpleasant, pleasant and neutral scenes. Brain activations are illustrated in Table 3 and Fig 4. For each emotional valence, the motivational task (compared to the emotional task) activated motor areas. However, in this task (compared to in the emotional task), activations in temporal regions (bilateral middle temporal gyrus) were only observed for pleasant and neutral scenes, and only pleasant scenes induced supplementary activation in the parietal cortex–in the left inferior and superior parietal gyri. As before, no significant activations were observed in the emotional task compared to the motivational task for each emotional valence.

**Table 3:** Cerebral regions specifically involved in emotional (ET) and motivational (MT) tasks depending on the emotional valence when tasks were contrasted against one another ([ET > MT] and [MT > ET]) for unpleasant, pleasant and neutral scenes.

Cerebral regions	[MT > ET]	
	(x,y,z)	k
**Temporal lobe**		
Middle temporal gyrus	L -56;-67;19	85
	L -58;-57;-5	37
	R 45;-47;28	66
**Parietal lobe**		
Angular gyrus	R 45;-75;31	27
Inferior parietal gyrus	L -46;-40;43	21
Precuneus	L/R 2;-50;58	45
**Frontal lobe**		
Middle frontal gyrus	L -46;8;52	57
Superior frontal gyrus	L -26;8;67	150

MNI coordinates (x, y, z), cluster extent (k = number of voxels) and laterality (Left-L and Right-R) were indicated for each activation cluster. Results were reported with a vowel-wise threshold of p<0.001 (uncorrected for multiple comparisons, T > 3.93) and a cluster extent threshold of 20 voxels.

**Fig 5:**
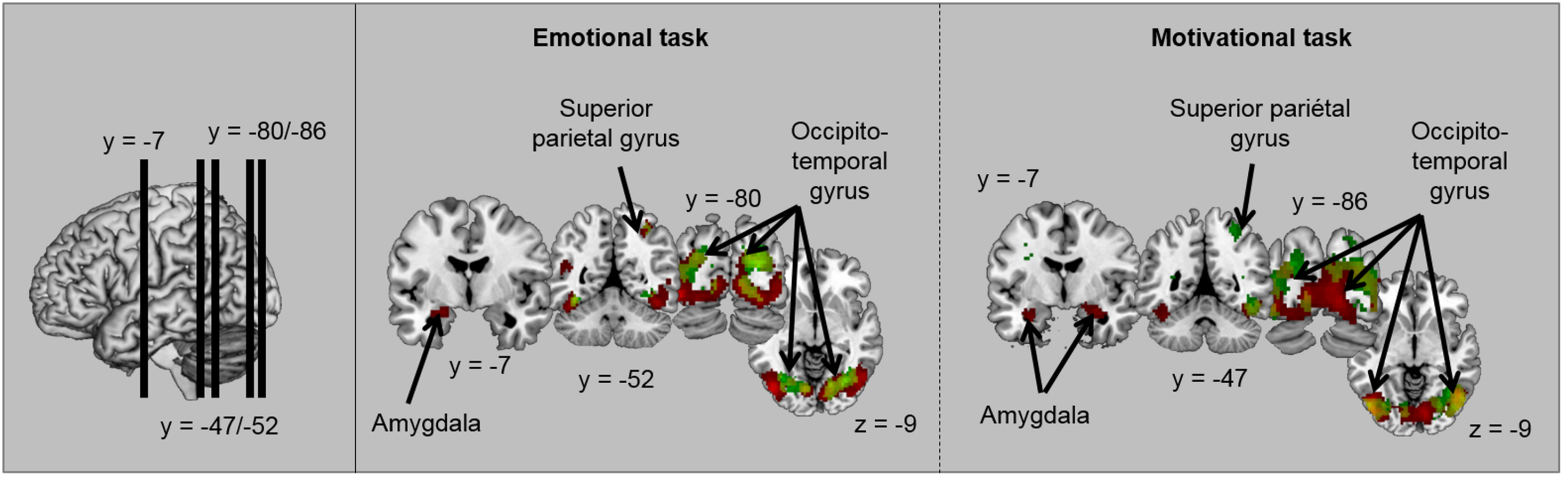
Illustration of activations obtained for the contrast [Unpleasant > Neutral] (in red), for the contrast [Pleasant > Neutral] (in green) and for both contrasts (in yellow) in each task.

Secondly, we identified the brain regions involved in the emotional processing of visual stimuli in each task by contrasting each category of emotional scene to neutral scenes for the emotional task ([ET Unpleasant > ET Neutral] and [ET Pleasant > ET Neutral]) and the motivational task ([MT Unpleasant (Avoiding) > MT Neutral (no action)] and [MT Pleasant (Approach) > MT Neutral (no action)]). Brain activations are illustrated in Table 4 and Fig 5. In both tasks, a higher level of activity was observed in bilateral fusiform gyrus for unpleasant scenes and pleasant scenes than for neutral scenes. Interestingly, results also revealed activation of the amygdala. For unpleasant scenes (versus neutral scenes), the amygdala was activated bilaterally in the motivational task and only in the right hemisphere in the emotional task.

**Table 4:** Cerebral regions specifically involved in emotional processing of visual scenes in each task obtained by contrasting each category of emotional scenes to neutral scenes for the emotional task ([ET Unpleasant > ET Neutral] and [ET Pleasant > ET Neutral]) and the motivational task ([MT Unpleasant (Avoiding) > MT Neutral (no action)] and [MT Pleasant (Approach) > MT Neutral (no action)]).

Cerebral regions	Emotional task				Motivational task			
	[Unpleasant>Neutral]		[Pleasant>Neutral]		[Unpleasant(Avoidance)> Neutral (no action)]		[Pleasant (Approach)> Neutral (no action)]	
	(x,y,z)	k	(x,y,z)	k	(x,y,z)	k	(x,y,z)	k
**Occipital and temporal lobes**								
Fusiform gyrus	L/R -46;-77;1	4025**	L 20;-85;19	886	L/R -46;-75;-11	4014**	L/R -46;-75;-11	267
			R -36;-62;-8	730				
**Parietal lobe**								
Superior parietal gyrus	R 30;-52;49	47					R 30;-52;55	132
Supramarginal gyrus							R 57;-22;28	26
**Frontal lobe**								
Precentral gyrus							L -46;-15;55	50
**Subcortical regions**								
Amygdala/Hippocampus	L -26;-7;-17	37			L -28;-2;-26	139		
					R 30;-5;-17	47		

MNI coordinates (x, y, z), cluster extent (k = number of voxels) and laterality (Left-L and Right-R) were indicated for each activation cluster. Results were reported with a vowel-wise threshold of p<0.001 (uncorrected for multiple comparisons, T > 3.93) and a cluster extent threshold of 20 voxels. Significant activities obtained after statistical correction for multiple comparisons (FEW) with a voxel-wise threshold of p<0.05 (**) and p<0.1(*) and a cluster extent of 10 voxels were also indicated.

#### Effect of spatial frequencies in each task

Finally, the influence of spatial frequency content in each task was assessed by contrasting the spatial frequency conditions against one another (NF, HSF, LSF) in the emotional and motivational tasks. Brain activations are illustrated in Table 5. Based on a previous behavioral study carried out by our team [1], we assumed that the emotional and motivational tasks would be preferentially based on the processing of HSF and LSF information respectively. We therefore expected to observe higher activations for HSF scenes in the emotional task, and for LSF stimuli in the motivational task. Results of the present study showed that the HSF scenes elicited greater activation in the fusiform gyrus than LSF stimuli in both the emotional and motivational tasks (it should be noted that stronger and bilateral activations were observed for the motivational task). Stronger activations in the fusiform gyrus were also observed for NF scenes than for LSF scenes in both tasks. No significant activations were observed for LSF scenes compared to HSF and NF scenes in either task. These results highlight a prevalence of HFS information (in HSF and NF stimuli, compared to LSF information) in visual areas in both tasks, irrespective of emotional valence.

**Table 5:** Influence of spatial frequency content on activated cerebral regions in each task.

Cerebral regions	Emotional task						Motivational task					
	[HSF> LSF]		[NF > HSF]		[NF > LSF]		[HSF > LSF]		[NF > HSF]		[NF > LSF]	
	(x,y,z)	k	(x,y,z)	k	(x,y,z)	k	(x,y,z)	k	(x,y,z)	k	(x,y,z)	k
**Occipital lobe**												
Fusiform gyrus			L -21;-62;1	98	L -31;-50;-14	33	L -28;-72;-14	916**			L -3;-87;-2	56
	R 15;-95;-6	114					R 20;-92;-8	852				
	R 35;-52;11	143	R 17;-50;4	28								
**Temporal lobe**												
Parahippocampal gyrus											R 32;-37;-11	92
Middle temporal gyrus									L 57;-60;22	27		

Activations obtained by contrasting the spatial frequency conditions (NF, HSF, LSF) in the emotional task and the motivational task. Only significant contrasts are shown in Table 2. MNI coordinates (x, y, z), cluster extent (k = number of voxels) and laterality (Left-L and Right-R) were indicated for each activation cluster. Results were reported with a vowel-wise threshold of p<0.001 (uncorrected for multiple comparisons, T > 3.93) and a cluster extent threshold of 20 voxels. Significant activities obtained after statistical correction for multiple comparisons (FEW) with a voxel-wise threshold of p<0.05 (**) and a cluster extent of 10 voxels were also indicated.

In order to assess how spatial frequency content influenced each task depending on the emotional valence of visual scenes, the contrasts between spatial frequency conditions described above were replicated by distinguishing unpleasant, pleasant and neutral scenes in each task. Brain activations are illustrated in Table 6. Overall, in each task, we confirmed that the fusiform gyrus cortex is more strongly activated by HSF than by LSF scenes for each emotional valence, except during the emotional categorization of neutral scenes (it should be noted that activated clusters were again larger in the motivational task). Other weak activations were reported for HSF scenes (in comparison to LSF scenes), mainly in response to unpleasant scenes in the emotional task. Activated regions included the superior parietal gyrus, the middle frontal cortex and some subcortical nuclei (putamen and caudate nucleus).

**Table 6:** Influence of spatial frequency content on activated cerebral regions in each task depending on emotional valence.

Cerebral regions	Emotional task						Motivational task					
	[HSF>LSF]						[HSF>LSF]					
	Unpleasant		Pleasant		Neutral		Unpleasant (Avoidance)		Pleasant (Approach)		Neutral (no action)	
	(x,y,z)	k	(x,y,z)	k	(x,y,z)	k	(x,y,z)	k	(x,y,z)	k	(x,y,z)	k
**Occipital lobe**												
Fusiform gyrus	L/R 5;-90;-11	37					L/R 25;-92;-6	176	L 25;-90;13	353	L -23;-85;-11	134
	R -33;-85;37	37	R 17;-97;1	28			L -13;-90;-14	178	R -21;-90;10	241	R 22;-92;7	202
									R 20;-60;55	59		
**Parietal lobe**												
Superior parietal gyrus	L -26;-65;40	27							R 20;-60;55	59		
**Frontal lobe**												
Inferior frontal gyrus					R 45;18;19	35						
Middle frontal gyrus	L -33;11;31	24										
Superior frontal gyrus					L -13;60;28	33						
**Subcortical regions**												
Putamen	R 25;-5;10	24										
Caudate nucleus	L -13;-10;25	24										

Activations obtained by contrasting the spatial frequency conditions (HSF and LSF) against one another in each task for unpleasant, pleasant and neutral scenes. MNI coordinates (x, y, z), cluster extent (k = number of voxels) and laterality (Left-L and Right-R) were indicated for each activation cluster. Results were reported with a vowel-wise threshold of p<0.001 (uncorrected for multiple comparisons, T > 3.93) and a cluster extent threshold of 20 voxels. (b) Illustration of activations obtained by the contrast [MT > ET] for each emotional valence.

**Fig 6:**
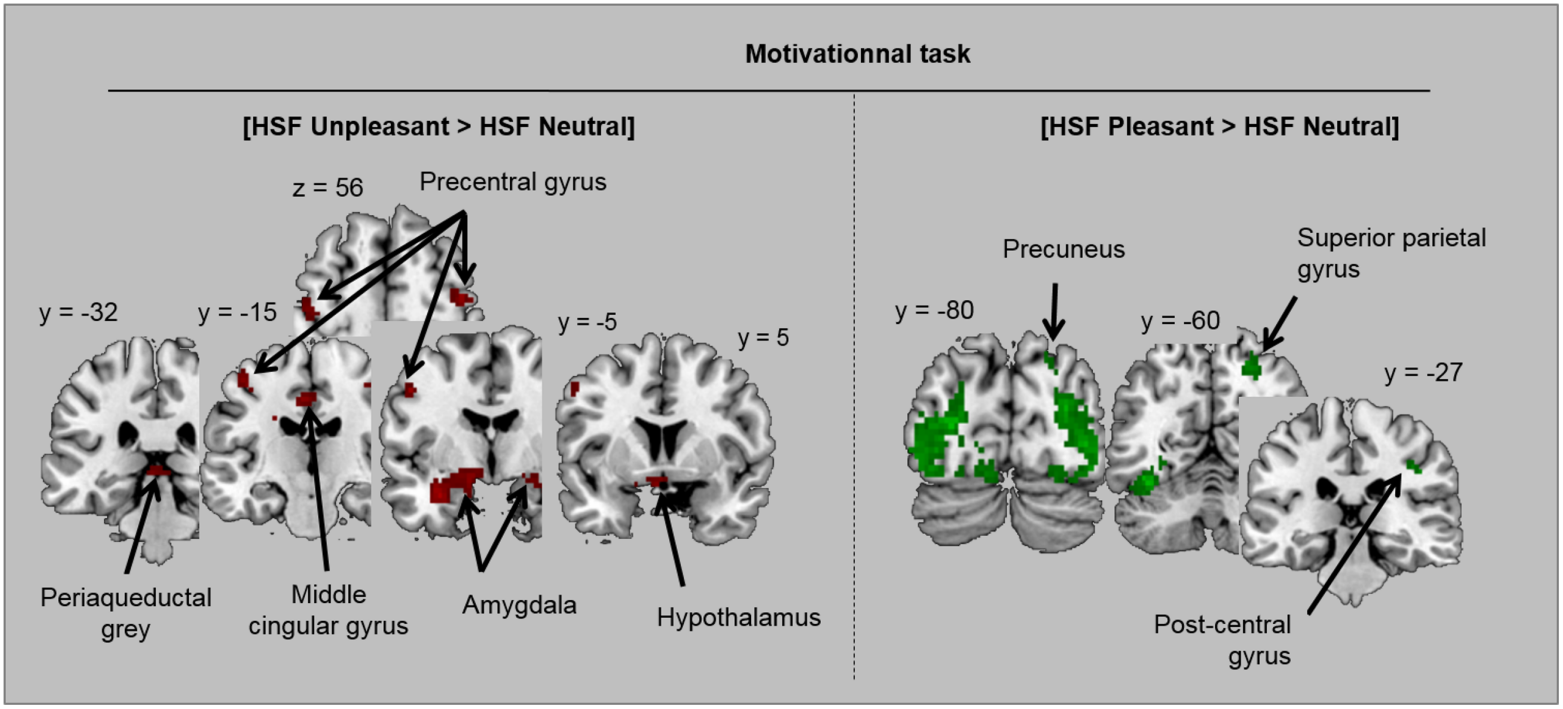
Illustration of activations obtained in the motivational task for the contrasts ([HSF Unpleasant> HSF Neutral] and [HSF Pleasant > HSF Neutral]).

Finally, in order to assess how spatial frequencies influenced emotional processing in each task, we contrasted each category of emotional scene (unpleasant and pleasant) to neutral scenes by distinguishing NF, HSF and LSF scenes. Brain activations are illustrated in Tables 7 and 8 and Fig 6. In the emotional task, and in comparison to neutral scenes, unpleasant scenes induced significant activation in bilateral fusiform gyrus for each spatial frequency, and in parietal regions only for HSF scenes (superior parietal gyrus) and NF scenes (supra-marginal gyrus and precuneus). For pleasant scenes in comparison to neutral scenes, only scenes containing LSF (LSF or NF scenes) elicited significant activations in the emotional task. Activated regions included bilateral fusiform gyrus (for LSF and NF scenes) and the superior frontal gyrus (for LSF scenes). In the motivational task, both unpleasant and pleasant scenes (each in comparison to neutral scenes) induced higher activity in bilateral fusiform gyrus for each spatial frequency. Additional activations were observed specifically for HSF scenes, and differed according to the valence of scenes. For pleasant HSF scenes (versus neutral HSF scenes), activated regions included parietal areas (also activated for NF scenes) and the superior frontal gyrus. For unpleasant HSF scenes (compared to neutral HSF scenes), they included i) a cortical network, comprising frontal areas—motor areas and the superior frontal gyrus—and the middle cingular gyrus, and ii) a subcortical network including the amygdala and hippocampal complex, the hypothalamus and the periaqueductal grey.

**Table 7:** Cerebral regions specifically involved in emotional processing of visual scenes ([Unpleasant > Neutral] and [Pleasant > Neutral]) for each spatial frequency content (LSF, HSF, NF) during the emotional task.

Cerebral regions	Emotional task											
	[Unpleasant > Neutral]						[Pleasant > Neutral]					
	LSF		HSF		NF		LSF		HSF		NF	
	(x,y,z)	k	(x,y,z)	k	(x,y,z)	k	(x,y,z)	k	(x,y,z)	k	(x,y,z)	k
**Occipital and temporal lobes**												
Fusiform gyrus	L -48;-80;1	528**	L/R -48;-77;-2	3079**	L/R -33;-75;22	2138**	L -33;-82;10	265				
	R 45;-72;-8	518**					R 35;-70;-5	304			R 30;-55;-11	74
**Parietal lobe**												
Superior parietal gyrus			R 30;-52;49	46								
Supramarginal gyrus					L -56;-50;28	39						
Precuneus					L -6;-50;37	29						
**Frontal lobe**												
Superior frontal gyrus							L -11;51;1	27				
							R 12;61;7	28				

Results were reported with a vowel-wise threshold of p<0.001 (uncorrected for multiple comparisons, T > 3.93) and a cluster extent threshold of 20 voxels. Significant activities obtained after statistical correction for multiple comparisons (FEW) with a voxel-wise threshold of p<0.05 (**) and a cluster extent of 10 voxels were also indicated.

**Table 8:** Cerebral regions specifically involved in emotional processing of visual scenes ([Unpleasant > Neutral] and [Pleasant > Neutral]) for each spatial frequency content (LSF, HSF, NF) during the motivational task.

Cerebral regions	Motivational task											
	[Unpleasant (Avoidance) > Neutral (no action)]						[Pleasant (Approach) > Neutral (no action)]					
	LSF		HSF		NF		LSF		HSF		NF	
	(x,y,z)	k	(x,y,z)	k	(x,y,z)	k	(x,y,z)	k	(x,y,z)	k	(x,y,z)	k
**Occipital and temporal lobes**												
Fusiform gyrus	L -48;-77;-8	458*	L/R -46;-80;1	3029**	L/R -46;-72;-8	2122**	R 27;-82;4	307	L -36;-62;-17	1001**	L -28;-72;-11	437
	R 50;-62;-11	850**					L -48;-77;1	20	R 47;-62;-11	1151**	R 32;-77;13	234
							L -31;-70;-2	38				
**Parietal lobe**												
Middle cingular gyrus			L/R -6;-20;43	70								
Superior parietal gyrus									R 22;-60;55	52	R 27;-47;61	35
Precuneus									R 20;-77;43	26		
Post-central gyrus									R 42;-25;34	21		
**Frontal lobe**												
Superior frontal gyrus			L/R 2;63;13	72					L/R -1;66;16	28		
			L/R -16;56;31	47								
Precentral gyrus			L -48;-5;40	36								
			L -43;-17;55	34								
			R 42;-12;55	49								
**Subcortical regions**												
Amygdala/Hippocampus			L -33;-7;-23	171								
Hypothalamus			R 25;-5;-14	29								
Periaqueductal grey substance			L/R -3;-32;-2	23								

Results were reported with a vowel-wise threshold of p<0.001 (uncorrected for multiple comparisons, T > 3.93) and a cluster extent threshold of 20 voxels. Significant activities obtained after statistical correction for multiple comparisons (FEW) with a voxel-wise threshold of p<0.05 (**) and a cluster extent of 10 voxels were also indicated.

#### ROIs analyses in the amygdala

**Fig 7:**
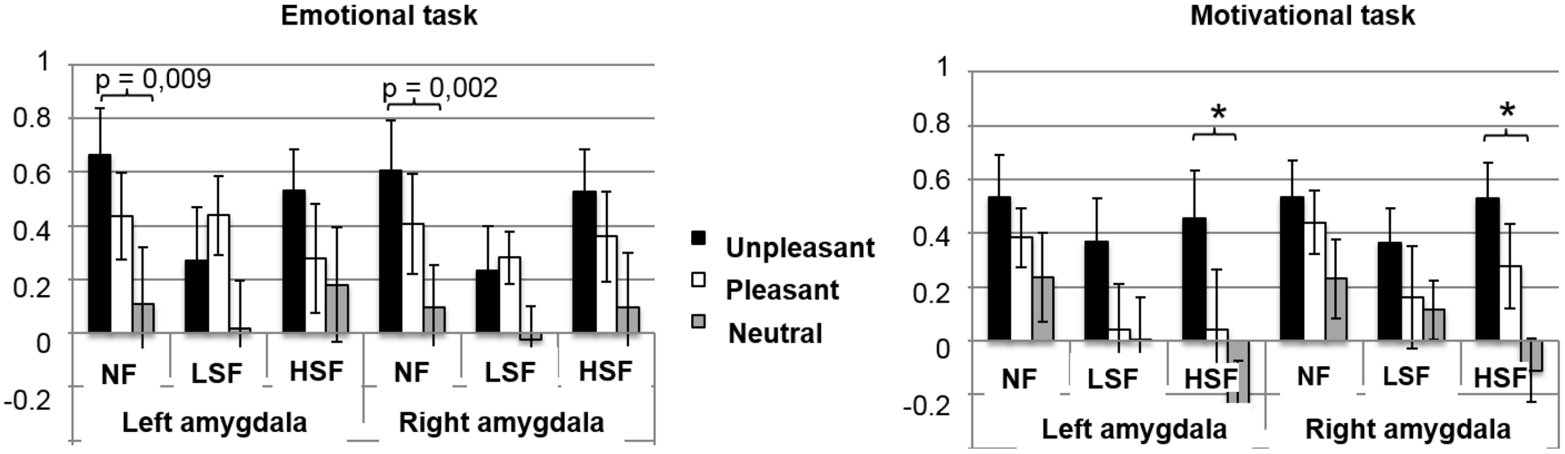
ROIs analyses in amygdala. Mean percentage of signal change in the right and left amygdala in each condition depending on the emotional valence (unpleasant, pleasant, neutral) and the spatial frequency content of scenes (NF, LSF, HSF). * p<0.001.

ROI analysis in the right and left amygdala revealed that amygdala activity was modulated statistically only by Emotional valence (left amygdala: F1,12 = 10.02; p < 0.001; η2 = 0.45; right amygdala: F1,12 = 12.62; p < 0.001; η2 = 0.51). Higher levels of activity in the left and right amygdala were obtained for unpleasant scenes than for neutral scenes (left amygdala: F1,12 = 17.73; p < 0.001; right amygdala: F1,12 = 38.91; p < 0.001). No significant difference was found between pleasant and neutral scenes for the left (F1,12 = 5.64; p = 0.03) or right amygdala (F1,12 = 7.60; p < 0.02). Given our assumptions, the effect of emotional valence on amygdala activity was explored according to the task and spatial frequency content using planned comparisons (see Fig 7). Planned comparisons showed that in the emotional task (ET), NF unpleasant scenes (compared to NF neutral scenes) activated the right amygdala more strongly (right amygdala: F1,12 = 15.24; p = 0.002; left amygdala: F1,12 = 9.49; p = 0.009), whereas in the motivational task (MT), unpleasant HSF scenes (compared to neutral HSF scenes) elicited greater activation in both the right (F1,12 = 19,22; p < 0,001) and left amygdalae (F1,12 = 17,15; p < 0,001). However, no significant difference was found in either task between pleasant and neutral scenes in either amygdala, irrespective of the spatial frequency content of scenes. Results appear in Fig 7.

## Discussion

The first goal of this pilot fMRI study was to identify the cerebral correlates involved in the visual processing of emotional scenes during two affective tasks: one emotional, based on the appraisal of a personal emotional experience, and the other motivational, based on the appraisal of the tendency to action. Secondary, we investigated the effect of the type of spatial frequency information on cerebral activity in each task.

### Brain structures involved in emotional and motivational tasks

#### Appraisal-specific networks according to task

Firstly, we identified the brain regions specifically involved in the emotional task based on emotional experience, and in the motivational task based on action tendency. Overall, contrasts between the two tasks showed that the motivational task induced greater activity in occipito-temporal visual regions than the emotional task. This result suggests that the visual perceptual processing regions are more heavily recruited by appraisal of the tendency to action than by appraisal of personal emotional experiences. However, this effect should be interpreted in correlation with behavioral performances. Participants responded more slowly in the motivational task than in the emotional task. These results suggest that appraisal of the tendency to action is more difficult than appraisal of emotional valence. These results differ from our previous behavioral results [1] which showed that the motivational task is carried out more quickly than the emotional task. It is possible that in our fMRI study the position of participants during the experiment, i.e. lying down (versus seated in the behavioral study) made performance of the motivational task more difficult.

Interestingly, our whole-brain analyses revealed activation of a specific brain network in the motivational task (compared to the emotional task). This included frontal regions involved in voluntary motor programming, such as the motor cortex (precentral gyrus) and the supplementary motor area (SMA) [80–82] and parietal regions involved in spatial perception, such as the bilateral precuneus [83], the superior and inferior parietal gyri [84–86] and the right angular gyrus [87]. In contrast, no significant activation was observed for the emotional task compared to the motivational task. Although the two tasks required the same type of motor response, the activation of motor and parietal areas during the motivational task suggests that in this task a particular form of motor programming is required to situate participants’ action in their personal space. Although participants did not perform a real action, they had to indicate the action they would adopt if they really were involved in the scene. Several studies have shown that mental imagery of actions induces activations related to motor programming similar to those of real actions [88–90]. Our study therefore indicates that the motivational task is well-suited to the exploration of voluntary motor behaviour associated with emotion.

Contrasts between the two tasks based on the emotional valence of stimuli showed that frontal activations associated with motor programming were mainly observed for pleasant scenes (tendency to approach) and for unpleasant scenes (tendency to avoidance). Parietal activations (inferior and superior parietal gyri) were relatively specific to the tendency to approach. The inferior and superior parietal regions are known to be involved in goal-directed spatial and non-spatial attention [91,92], in forming representations of body state and space [84,85,93–95] and motor imagery [88]. A recent study also reported the role of superior parietal regions in processing the visual context within an egocentric reference frame [86]. Based on this literature, the parietal activations obtained for the tendency to approach suggest that this behavior probably involves the processing of spatial relations between objects, and between objects and the individual performing the task, probably in order to facilitate mental navigation.

#### Emotion-specific networks according to task

Secondly, for each task, we contrasted both types of emotional scene (pleasant and unpleasant) to neutral scenes. Results showed that unpleasant and pleasant scenes activated a bilateral occipito-temporal network for both emotional and motivational tasks. Moreover, in both tasks, response times were shorter for emotional than for neutral scenes. Interpreted together, these behavioral and neuroimaging results suggest greater activation of visual processing regions in response to emotional scenes (unpleasant and pleasant) than to neutral stimuli, and this heightened activation could possibly facilitate appraisal mechanisms in both tasks [14,96–98]. Our results are consistent with previous studies using emotional tasks based on personal emotional experiences [5,7–9,14,24,53,99,100]. Interestingly, the result which shows that in emotional scenes, appraisal of the tendency to action involves greater recruitment of the visual regions (compared to neutral scenes) is original and explicitly supports the idea that the motivational system may modulate perceptual processing in order to facilitate selection of an appropriate action [33,101].

Furthermore, the modulation of activity in visual regions by emotional scenes could be mediated by the amygdala. The amygdala is involved in emotional processing and has direct and reciprocal connections with sensory areas including the visual cortices [10–16]. Our whole-brain analyses showed that only unpleasant scenes activated the amygdala in both emotional and motivational tasks. This result was confirmed by the ROI analysis, which showed right amygdala activation during the emotional task and bilateral amygdala activation during the motivational task, for unpleasant scenes only. No significant amygdala activation was found for pleasant scenes during either task. Our results are in agreement with other data showing that the right amygdala plays a critical role in the appraisal of the emotional salience of unpleasant stimuli [53], particularly frightening ones [102]. More interestingly, amygdala involvement during the motivational task suggests that this region may also play a role in the choice of tendency to action, particularly of the defensive type. Human behavior is mainly based on two motivational systems, appetitive and aversive, which are sub-served by distinct networks [103]. The aversive system may particularly engage the amygdala in relation to unpleasant affects and defensive behaviors. Our results provide tangible evidence of this close relationship between bilateral amygdala activity and the development of defensive behaviors in relation to unpleasant scenes. In contrast, as mentioned previously, there was no activation of the amygdala in response to pleasant stimuli either in the emotional task or in the motivational task, suggesting that this structure does not play a major role in the appraisal of pleasant experiences or in the tendency to approach. According to some authors, activation of the amygdala is related more to the arousal level induced by stimulus rather than to its emotional valence [104]. This idea is supported by other studies showing similar activation of the amygdala in response to pleasant and unpleasant stimuli matched for arousal [66,105,106]. In our study, the arousal level induced by pleasant scenes was significantly lower than the level measured for unpleasant scenes. Based on previously related studies, it is possible that this arousal level was perhaps not high enough to induce significant activation of the amygdala in either task.

Finally, in the emotional task, additional activation of the superior parietal cortex appeared in response only to unpleasant scenes. Given the role of the superior parietal cortex in both emotional processing and action-related functions [84,86,94,95], this result supports the hypothesis of a close relationship between emotional appraisal and action.

### Spatial frequency-specific networks according to task

The second aim of our study was to investigate the influence of the type of spatial frequency on the cerebral network involved in the visual processing of emotional scenes in each task.

#### Emotional task

In the emotional task, contrasts between the spatial frequency conditions (HSF, LSF, NF) showed that the bilateral visual occipito-temporal network recruited during this task was more strongly activated by scenes filtered in HFS than by scenes filtered in LSF or unfiltered scenes. When these contrasts were replicated and a distinction was made between each type of emotional valence, the HSF effect was obtained only for pleasant and unpleasant scenes, suggesting that the HSF effect is valence-specific in visual areas.

Moreover, the comparison of emotional (unpleasant or pleasant) to neutral scenes depending on spatial frequency recruited supplementary regions. Unpleasant scenes activated the bilateral fusiform gyrus for each spatial frequency (NF, HSF and LSF). It is worth noting that the activation cluster size was greater for the HSF scenes. In addition, only HSF unpleasant scenes (compared to HSF neutral scenes) induced significant activity in the superior parietal gyrus, which is known to be involved in tasks requiring a first-person point of view [83] as was the case in our emotional task. It appears here that the detailed information conveyed by HSF in unpleasant scenes is the most useful in the evaluation of personal experience of unpleasantness. However, this prevalence of HSF was not observed in our behavioral data, as only unpleasant NF scenes were categorized faster than unpleasant HSF scenes.

Pleasant scenes (compared to neutral scenes) activated fusiform gyrus and superior frontal region during the processing of scenes containing LSF (LSF or NF scenes) only. This result suggests that the coarse information conveyed by LSF in pleasant scenes is the most useful for the evaluation of personal experience of pleasantness. In addition, this result was supported by behavioral data. Participants responded more rapidly only to LSF pleasant scenes than to HSF pleasant scenes. To sum-up for the emotional task, when the aim is to identify a personal emotional experience, the activity of cerebral networks involved in the processing of emotional scenes appears to be modulated mainly by detailed HSF information for unpleasant scenes and by coarse LSF information (or NF) for pleasant scenes.

#### Motivational task

In the motivational task, a spatial frequency effect was observed only from fMRI data, which showed that activity in the visual occipito-temporal network was significantly greater for HSF scenes than for LSF scenes. This result was replicated for the three emotional valences (unpleasant, pleasant and neutral) when they were analyzed separately, suggesting that the effect of HSF we observed in visual areas was independent of the emotional content of stimuli.

Moreover, the comparison between emotional (unpleasant or pleasant) and neutral scenes according to spatial frequency recruited supplementary regions mainly for HSF scenes, when the content was both pleasant and unpleasant. Unpleasant HSF scenes (compared to neutral HSF scenes) induced significant activity in the mid-cingulate cortex, premotor cortex and superior frontal gyrus as well as in the amygdala, hypothalamus and periaqueductal grey matter. The mid-cingulate cortex plays a major role in the integration of cognitive and affective information in the control of action [107–111]. It is functionally connected to sensorimotor regions such as the premotor cortex [112] and receives inputs, directly and indirectly, from emotion-related cerebral regions such as the amygdala [108,113]. Regarding the hypothalamus and the periaqueductal grey matter, some studies have shown their involvement in “motor vigilance” (as proposed by [114]) and in the development of defensive behaviors [115,116]. Based on this literature, our results suggest that the implementation of defensive behaviors during unpleasant scene processing in the motivational task involves coordination between the voluntary motor system and a “motor vigilance” system. They also suggest that this coordination comes under the control of cognitivo-affective activity in the middle cingular gyrus and the amygdala and is based on the fine and detailed information conveyed by HSF in scenes.

Significant activations were measured within the superior parietal cortex for pleasant HSF scenes (compared to neutral HSF scenes). Given the role of the parietal cortex in navigation and an individual’s projection of his/her body into the space it occupies, our results suggest the importance of the fine and detailed information conveyed by HFS when approaching in the context of a pleasant scene. To sum-up for the motivational task, our results suggest that when the aim is to evaluate action tendencies, the activity of cerebral networks involved in processing emotional scenes is based predominantly on detailed HSF information for unpleasant and pleasant scenes. This prevalence of HSF in the motivational task could come as a surprise in view of previous studies which suggest that processing of the action tendency depends more on LSF [60,61]. In addition, our behavioral data showed no effect of spatial frequency information during the motivational task. This last result is somewhat different from our previous behavioral results [1] where we reported that LSF information was required for rapid identification of the tendency to action. An explanation of the discrepancy between results may be related to the position participants had to adopt during the experiments, (lying down in the present study versus seated in our previous study). Indeed, this position influences both actions and motor representations of the body [117]. The supine position in the MR scanner might have influenced the processing of spatial frequency information and specifically favored the use of HSF information during the motivational task. Finally, although participants were instructed to respond as quickly as possible, their choice of action and the time taken to make their decision had no consequences for them. In a real situation, the choice of a prompt and appropriate action may be necessary when an individual is confronted with situations such as those featured in the present study (especially unpleasant scenes). In this perspective, one study by [49] suggests that confronting participants with the consequences of their actions during emotional processing would favor the use of LSF in the processing of emotional information. These remaining questions and discrepancies should be studied in a future work.

## Conclusions

Results showed a larger cerebral network in the motivational task based on the appraisal of the tendency to action than in the emotional task based on the appraisal of the personal emotional experience, although the rating of emotional and motivational dimensions were closely correlated in our study. In both tasks, activations were found in the visual areas of gyrus fusiform for all emotional scenes and in the amygdala for unpleasant scenes only. The motivational task induced additional activations in frontal motor-related areas for unpleasant scenes (tendency to avoidance) and in navigation-related areas for pleasant scenes (tendency to approach). Furthermore, the cerebral activity in the emotional task was modulated mainly by HSF for unpleasant scenes and by LSF (or NF) for pleasant scenes. Cerebral activity in the motivational task was driven mainly by the HSF contained in scenes for unpleasant and pleasant scenes. However, given the complexity of the experimental design, these preliminary results need to be confirmed in future work involving a larger number of participants. Moreover, it should be noted that the emotional and motivational dimensions were combined in the stimuli selected for our study. Unpleasant stimuli systematically induce a tendency to avoid, while pleasant stimuli systematically induce a tendency to approach. It is possible that additional activations specific to each task might appear if stimuli which distinguish clearly between the two dimensions–valence and tendency to action—were used. This hypothesis should be explored in future research. The present pilot study establishes a distinction between the visual processing of emotional scenes during identification of the tendency to action, and visual processing during identification of a personal emotional experience. It illustrates, moreover, the flexible use of spatial frequencies contained in scenes depending on their emotional valence and on task demands.
